# A comparative study of external female genitalia (including the 8 ^th^ and 9 ^th^ abdominal segments) in the family Megalopodidae and other related families of Chrysomeloidea

**DOI:** 10.3897/zookeys.762.22163

**Published:** 2018-05-30

**Authors:** Kaiqin Li, Hongbin Liang

**Affiliations:** 1 Key Laboratory of Zoological Systematics and Evolution, Institute of Zoology, Chinese Academy of Sciences, Beijing 100101, China; 2 Kunming Natural History Museum of Zoology, Kunming Institute of Zoology, Chinese Academy of Sciences, Kunming 650223, China

**Keywords:** Chrysomeloidea, female genitalia, Megalopodidae, morphology, 8^th^ abdominal segment

## Abstract

The external female genitalia of 29 species belonging to three genera of Megalopodidae and 80 species belonging to 61 genera of another four families in Chrysomeloidea were studied. The external female genitalia within the superfamily Chrysomeloidea can be divided into a cerambycid type and a chrysomelid type. The comparative study of external female genitalia shows Megalopodidae is more closely related to the family Cerambycidae than to the family Chrysomelidae *s.l.* Among five subfamilies of Cerambycidae we studied, the subfamily Lamiinae is most closely allied to Megalopodidae. An evolutionary path is proposed for the spiculum gastrale in Chrysomeloidea: the characteristic state of the spiculum gastrale without a joint is primary, and that with a joint is secondary. The family Orsodacnidae has probably evolved in isolation from the early chrysomelids, due to their shared external female genitalia (cerambycid type). In the family Chrysomelidae, the structure of external female genitalia and ovipositing behavior show that the subfamily Synetinae is closer to the Camptosomata than the subfamily Eumolpinae. In general, the shape of the terminal ovipositor is palp-like in the Chrysomeloidea. Terminal ovipositors are generally palp-shaped in Chrysomeloidea except for those that are lamellate in the genus *Callispa* and the subfamily Cassidinae who produce egg-sheaths.

## Introduction

Adults of the family Megalopodidae commonly feed on the juices in stems and leaf tissues of plants in the families of Rosaceae, Oleaceae, Salicaceae, and Celastraceae, or consume the pollen of the *Araucaria* species (Tan et al. 1980, Reid 1989, Kuschel and May 1990, Li et al. 2013). They are distributed in Neotropical, Afrotropical, Palaearctic, Oriental, Nearctic and Australian regions (Tan et al. 1980). In China the Megalopodidae is represented by two subfamilies Megalopodinae and Zeugophorinae (Yu and Liang 2002, Medvedev 2010, Silfverberg 2010). The subfamily Megalopodinae includes two genera (*Temnaspis* Lacordaire, 1845 and *Poecilomorpha* Hope, 1840). The subfamily Zeugophorinae includes only a single genus (*Zeugophora* Kunze, 1818) which is divided into two subgenera, *Zeugophora* Kunze, 1818 and *Pedrillia* Westwood, 1864 (Monrós 1959, Gressitt and Kimoto 1961, Chen and Pu 1962, Kimoto and Gressitt 1979, Medvedev 1985, 1997, Reid 1989, 1992, 1995, Schöller 2009, Warchałowski 2010). There are about 450 species of Megalopodidae in the world with 31 species of Megalopodinae and 24 species of Zeugophorinae in China (Gressitt and Kimoto 1961, Chen and Pu 1962, Chen 1974, Kimoto and Gressitt 1979, Tan et al. 1980, Medvedev and Sprecher-Uebersax 1997, Medvedev 2002, 2010, Yu and Liang 2002, Medvedev 2010, Silfverberg 2010, Lawrence and Ślipiński 2013, Li et al. 2013). Latreille (1802) established the subfamily Megalopodinae. Chapuis (1874) constructed the early taxonomic system for the Chrysomelidae. The subfamily Megalopodinae was included in the Camptosomes for some time until Chen (1940) transferred this subfamily from the Camptosomes to the Eupodes, which was regarded as the most primitive group. The subfamily Megalopodinae was then included in the family Donaciidae. This included the subfamilies Sagrinae, Donaciinae, Orsodacninae, Megascelinae, and Megalopodinae. The subfamily Zeugophorinae was established by Böving and Craighead (1931) and was included in the family Orsodacnidae based on characteristics of larvae. Following research on the external male genitalia within the subfamily Zeugophorinae, Chûjô (1952) pointed out that subfamily Zeugophorinae was more closely related to the Megalopodinae than other taxa in the Chrysomeloidea. Chen (1964, 1973) proposed that both subfamilies Megalopodinae and Zeugophorinae belonged to the criocerid line. The criocerid line also included the six following subfamilies: Sagrinae, Donaciinae, Criocerinae, Bruchidae, Orsodacninae and Synetinae. Crowson (1981) included the subfamily Zeugophorinae as a member of Megalopodidae. Therefore the Megalopodidae now included two subfamilies the Megalopodinae and the Zeugophorinae. Kuschel and May (1990) added a new subfamily, the Palophaginae. Some researchers continued to regard the Megalopodidae (subfamilies Megalopodinae, Zeugophorinae, Palophaginae) as an independent family or subfamily (Lawrence and Britton 1991, Suzuki 1994). In addition, Reid (1995) also supported the Megalopodidae as an independent family and gradually more researchers accepted this treatment (Hunt et al. 2007, Gómez-Zurita et al. 2007, 2008; Marvaldi et al. 2009, Löbl and Smetana 2010, McKenna et al. 2015). However, the phylogenetic relationships were not well resolved among the Chrysomeloidea (Reid 1995, 2000, Hunt et al. 2007, Gómez-Zurita et al. 2007, 2008, Marvaldi et al. 2009, McKenna et al. 2015). Chen (1985) examined several characteristics including head orientation, male genitalia, larval feeding habits, thorax, and wing-venation, and proposed that the Megalopodidae was related to the family of Cerambycidae. Suzuki (1988, 1994) considered that the Megalopodidae probably descended from lamiid-type ancestors after studying their internal reproductive systems and hind wing venation. Schmitt (1992) also considered the Megalopodidae to be closer to the Cerambycidae than to the Chrysomelidae. Reid (1995) revealed Megalopodidae as a monophyletic group, with Orsodacnidae and Chrysomelidae as its sister groups, but the evidence supporting a close relationship between Megalopodidae and Cerambycidae was insufficient. In recent years, molecular evidence has shown that the Megalopodidae is closest to the Cerambycidae or Oxypeltidae (Hunt et al. 2007, Gómez-Zurita et al. 2007, 2008, Marvaldi et al. 2009, McKenna et al. 2015).

Male and female genitalia are among the most important identification characteristics in the Megalopodidae and receive much attention from taxonomists. Chûjô (1952, 1953) studied the morphology of external male genitalia. Following his extensive analysis of the external male genitalia of the Megalopodinae and Zeugophorinae, Chûjô found similarities between the two subfamilies. Subsequently, taxonomists began to describe the male genitalia of the Megalopodidae when publishing descriptions of new species and when they constructed or analyzed molecular phylogenetic relationships within the Chrysomeloidea (Reid 1989, Kuschel and May 1990, Mann and Crowson 1996, Verma 1996, Medvedev 1997).

Research on external female genitalia in the Megalopodidae is rare. There were only a few descriptions of male or female genitalia for this family when new species were described or revisions were made (Reid 1989, 1992, Li et al. 2013, Rodríguez-Mirón and Zaragoza-Caballero 2017). Suzuki (1994) reported comprehensively on the internal reproductive system of Chrysomeloidea *s.l.*, including the Megalopodinae and Zeugophorinae. Kasap and Crowson (1985) described briefly the 8^th^ abdominal segment of the external female genitalia of *Sphondylia afra* (Megalopodinae) and *Zeugophora fulvicollis* (Zeugophorinae). Reid (1989, 1992) described the structure of the ovipositor and spermathecae of the Zeugophorinae when he published new species. Recently Rodríguez-Mirón et al. (2017) studied the spermathecae morphology of the Megalopodidae. In the present study, we dissected and describe the 8^th^ abdominal segments and the external genitalia in females, then compared them with those of other related families in the Chrysomeloidea.

## Material and methods

### Preparation of specimens

All measurements were made with the aid of an ocular micrometer in the Nikon SMZ1500 stereoscopic microscope. Dry specimens were soaked in boiled water for 1–2 hours. For larger specimens the lateral margin of the abdomen was opened at its apex and the genitalia were pulled out of the abdomen with fine forceps. For smaller specimens the whole abdomen was removed from the body. The genitalia and/or whole abdomen were soaked in a warm solution of 10% KOH for 10–20 minutes as a pretreatment. The treatment time depended upon the degree of sclerotization found in different species. After pretreatment these organs were washed in water several times. Genitalia were then detached and some were dyed with Chlorazol Black E to stain membranous tissue a blue color. They were then transferred to glycerin for observation, photography, and preservation.

Photographs of female genitalia were taken using a Nikon SMZ-1500 stereoscopic dissecting microscope fitted with a Cannon 450D digital camera. Photographs of habitus were captured by a Cannon Macro 100 mm lens fitted to the Cannon 450D camera. For each final image, several photographs were taken at different focal planes, combined with Helicon Focus software to obtain one synthesized photograph, and finally edited with Adobe Photoshop software.

### Terminology

Morphological terminology for the female genitalia of the Megalopodidae follows Snodgrass (1935), Chûjô (1952, 1953), Kasap and Crowson (1985) and Lawrence et al. (2010). In females of the Chrysomeloidea, the genitalia segment is located on the 9^th^ segment of the abdomen. The ovipositor consists of the proctiger, paraproct, median plate, valvifer, coxite, and stylus (Kasap and Crowson, 1985). In contrast, the ovipositor of the Cerambycidae consists of the proctiger and vaginal palpi with baculi. The dorsal side of the baculi of the vaginal palpi is called the dorsal baculi. The ventral side of the baculi includes the paraproct baculi, valvifer baculi, and coxite baculi (Saito 1989). The ovipositor of the Megalopodidae includes two long vaginal palpi, which have one or two pair of baculi. Therefore, the ovipositor consists of the paraproct, valvifer, coxite, and stylus without a proctiger or a median plate.

Outside the ovipositor of *Nupserha bicolor* Thomson (Cerambycidae) there is a membranous bag-like capsule known as the capsular sheath (Dutt 1958). Saito (1989) suggested that this structure was the inter-segmental membrane found between the ovipositor and the 8^th^ segment. The ovipositor of Megalopodidae is surrounded by a pocket-like membrane. Kuschel and May (1990) named this membrane as a genital pocket. The base of the pocket is connected with the apical margin of the 8^th^ segment and the apex of the pocket is connected with the base of the ovipositor. The surface of the genital pocket is thickened by many sclerotized rings (Figs 9, 10).

### Specimens studied

Twenty-nine species belonging to three genera of Megalopodidae (including two subfamilies Megalopodinae and Zeugophorinae) were examined in this study (Table 1). Only one species *Orsodacne cerasi* (Linnaeus) is native to China. Twenty species belonging to 17 genera of five subfamilies (Prioninae, Spondylidinae, Lepturinae, Cerambycinae, and Lamiinae) were selected in the family Cerambycidae. Fifty-nine Chinese species in the Chrysomelidae belonging to 43 genera of 12 subfamilies (Table 1) were chosen including the Sagrinae, Bruchinae, Donaciinae, Criocerinae, Eumolpinae, Lamprosomatinae, Cryptocephalinae, Synetinae, Chrysomelinae, Galerucinae, Alticinae, and Cassidinae.

**Table 1.** Species of Chrysomeloidea studied.

Family	Subfamily	Species	Locality
Megalopodidae	Megalopodinae	*Poecilomorpha cyanipennis* (Kraatz)	Zhejiang (Hangzhou)
		*Poecilomorpha discolineata* (Pic)	Yunnan
		*Poecilomorpha downesi* (Baly)	Sichuan
		*Poecilomorpha laosensis* (Pic)	Indochina
		*Poecilomorpha maculata* (Pic)	Guangxi, Yunnan; Vietnam
		*Poecilomorpha mouhoti* (Baly)	Yunnan
		*Poecilomorpha pretiosa* Reineck	Guangxi, Zhejiang
		*Temnaspis bonneuili* Pic	Northeast of China
		*Temnaspis femorata* (Gressitt)	Fujian, Guangxi
		*Temnaspis fraxini* (Komiya)	Taiwan
		*Temnaspis humeralis* Jacoby	Hubei, Chongqing
		*Temnaspis nigriceps* Baly	Yunnan
		*Temnaspis japonica* Baly	Japan
		*Temnaspis nankinea* (Pic)	Henan
		*Temnaspis omeiensis* (Gressitt)	Sichuan
		*Temnaspis pulchra* Baly	Jiangxi
		*Temnaspis septemmaculata* (Hope)	Yunnan
		*Temnaspis syringa* Li and Liang	Beijing
		*Temnaspis vitalisi* (Pic)	Yunnan
	Zeugophorinae	Zeugophora (Pedrillia) annulata Baly	Heilongjiang
		Zeugophora (Pedrillia) bicolor Kraatz	Liaoning
		Zeugophora (Pedrillia) longicornis Westwood	Yunnan
		Zeugophora (Pedrillia) dimorpha (Gressitt)	Hunan
		Zeugophora (Pedrillia) yunnanica Chen and Pu	Yunnan
		*Zeugophora ancora* Reitter	Ningxia
		*Zeugophora cribrata* Chen	Qinghai
Megalopodidae	Zeugophorinae	*Zeugophora cyanea* Chen	Qinghai
		*Zeugophora scutellaris* Suffrian	Heilongjiang
		*Zeugophora turneri* Power	Beijing
Cerambycidae	Prioninae	*Aegolipton marginalis* (Fabricius)	China
		Megopis (Aegosoma) sinica sinica (White)	China
	Spondylidinae	*Asemum amurense* Kraatz	China
		*Spondylis buprestoides* (Linnaeus)	China
	Lepturinae	*Leptura annularis annularis* Fabricius	Heilongjiang
		*Leptura* sp.	Guangxi
		*Gaurotes virginea aemula* (Mannerheim)	China
	Cerambycinae	*Anoplistes halodendri* (Pallas)	Inner Mongolia
		*Aromia bungii* (Faldermann)	China
		*Purpuricenus temminckii* (Guérin-Méneville)	China
	Lamiinae	*Bacchisa comata* (Gahan)	Hainan
		*Eodorcadion brandti* (Gebler)	China
		*Glenea centroguttata* Fairmaire	Xizang
		*Glenea pulchra* Aurivillius	China
		*Monochamus alternatus alternatus* Hope	Hubei
		*Moechotypa diphysis* (Pascoe)	China
		*Oberea formosana* Pic	China
		*Paraglenea fortunei* (Saundeas)	China
		*Phytoecia rufiventris* Gautier des Cottes	China
		*Thyestilla gebleri* (Faldermann)	China
Orsodacnidae	Orsodacninae	*Orsodacne cerasi* (Linnaeus)	Yunnan
Chrysomelidae	Sagrinae	Sagra (Sagra) femorata Drury	China
		Sagra (Sagrinola) mouhoti Baly	Yunnan
	Bruchinae	*Callosobruchus chinensis* (Linnaeus)	China
	Donaciinae	*Donacia clavipes clavipes* Fabricius	Beijing
		*Donacia longicornis* Jacoby	China
		*Donacia provostii* Fairmaire	Hebei
		*Donacia vulgaris vulgaris* Zschach	Hebei
		*Macroplea mutica* (Fabricius)	Mongolia
		*Plateumaris weisei* (Duvivier)	China
		*Plateumaris sericea* (Linnaeus)	China
		*Plateumaris socia* (Chen)	Northeast of China
		*Sominella longicornis* (Jacobson)	China
		*Sominella macrocnemia* (Fischer Von Waldheim)	Heilongjiang
	Criocerinae	*Crioceris quatuordecimpunctata* (Scopoli)	China
		*Lema coromandeliana* (Fabricius)	Yunnan
		*Lema nigricollis* Jacoby	Yunnan
		*Lilioceris cheni* Gressitt & Kimoto	Guangxi
Chrysomelidae	Criocerinae	*Lilioceris consentanea* Lacordaire	China
		*Lilioceris gibba* (Baly)	Jiangxi, Fujian
		*Oulema oryzae* (Kuwayama)	Heilongjiang
	Eumolpinae	*Abiromorphus anceyi* Pic	Hebei
		*Basilepta* sp.	China
		*Chrysochus chinensis* Baly	Jilin, Heilongjiang
		*Colasposoma dauricum* Mannerheim	Yunnan
		*Platycorynus parryi* Baly	Guangxi
		*Platycorynus* sp.	Yunnan
	Lamprosomatinae	*Oomorphoides yaosanicu*s (Chen)	Fujian
	Cryptocephalinae	*Cryptocephalus altaicus* Harold	Inner Mongolia
		*Cryptocephalus limbellus semenovi* Weise	Inner Mongolia
		*Aspidolopha bisignata* Pic	Jilin, Yunnan
		*Clytra laeviuscula* Ratzeburg	Jilin
		*Physosmaragdina nigrifrons* (Hope)	Guangxi
		*Chlamisus stercoralis* (Jacoby)	Guangxi
	Synetinae	*Syneta adamsi* Baly	Shanxi
	Chrysomelinae	*Ambrostoma quadriimpressum* (Motschulsky)	Heilongjiang
		*Agasta formosa* Hope	Yunnan
		*Chrysolina aurichalcea* (Mannerheim)	Guangxi
		*Chrysomela populi* Linnaeus	Harbin, Jilin
		*Gastrophysa atrocyanea* (Motschulsky)	Sichuan
		*Phratora bicolor* Gressitt & Kimoto	Sichuan
	Galerucinae	*Gallerucida* sp.	Yunnan
		*Medythia nigrobilineata* (Motschulsky)	Heilongjiang
		*Mimastra limbata* Baly	Yunnan
		*Morphosphaera cavaleriei* Laboissière	China
		*Oides tarsatus* (Baly)	Guangxi
		*Paleosephraria* sp.	Yunnan
	Alticinae	*Altica viridicyanea* (Baly)	Beijing
		*Podontia affinis* (Gröndal)	Yunnan
		*Podontia dalmani* Baly	Yunnan
		*Podontia lutea* (Olivier)	Guangxi
		*Hemipyxis* sp.	China
	Cassidinae	*Octodonta nipae* (Maulik)	Hainan
		*Lasiochila cylindrica* (Hope)	Yunnan
		*Dactylispa* sp.	Yunnan
		*Callispa brettinghami* Baly	Yunnan
		*Callispa fortunei* Baly	Yunnan
		*Callispa nigricollis* Chen & Yu	Yunnan
		*Leptispa longipennis* (Gestro)	China
		*Basiprionota bisignata* (Boheman)	Guangxi

## Results

### Megalopodidae

**Figures 1–3.**
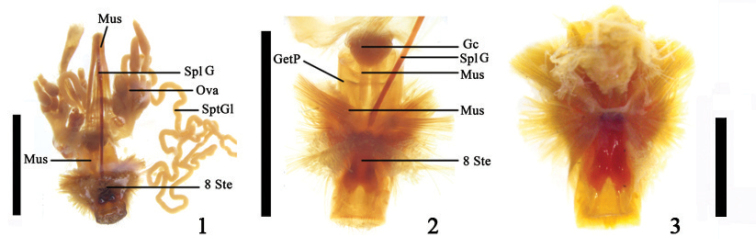
**1** Female internal reproductive system of *Temnaspis nankinea* (Pic), ventral view **2** 8^th^ and 9^th^ abdominal segment of *T. nankinea*, ventral view **3** Muscles of abdominal segment of *T. nankinea*, ventral view; Abbreviations: genital chamber (Gc); genital pocket (GetP); muscle (Mus); ovary (Ova); spermathecal gland (SptGl); spiculum gastrale (SplG); sternite 8 (8 Ste); scale line = 2.0 mm (Figs 1–2); scale line = 0.5mm (Fig. 3).

**Figures 4–6.**
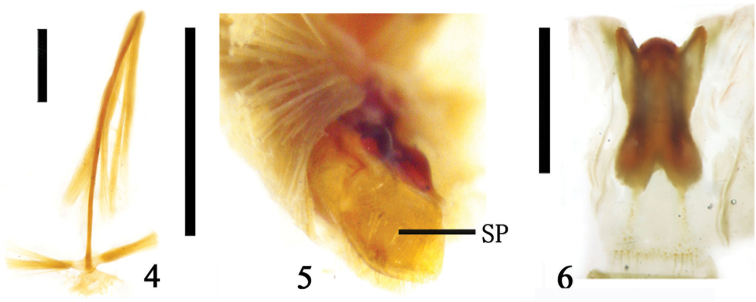
**4** Muscles of the spiculum gastrale of *T. nankinea*, ventral view **5** The apical sclerite of sternite 8 of *T. nankinea*, ventrolateral view **6** The genital plate of *T. nankinea*; Abbreviations: sclerotized plate (SP); scale line = 0.5 mm.

**Figures 7–12.**
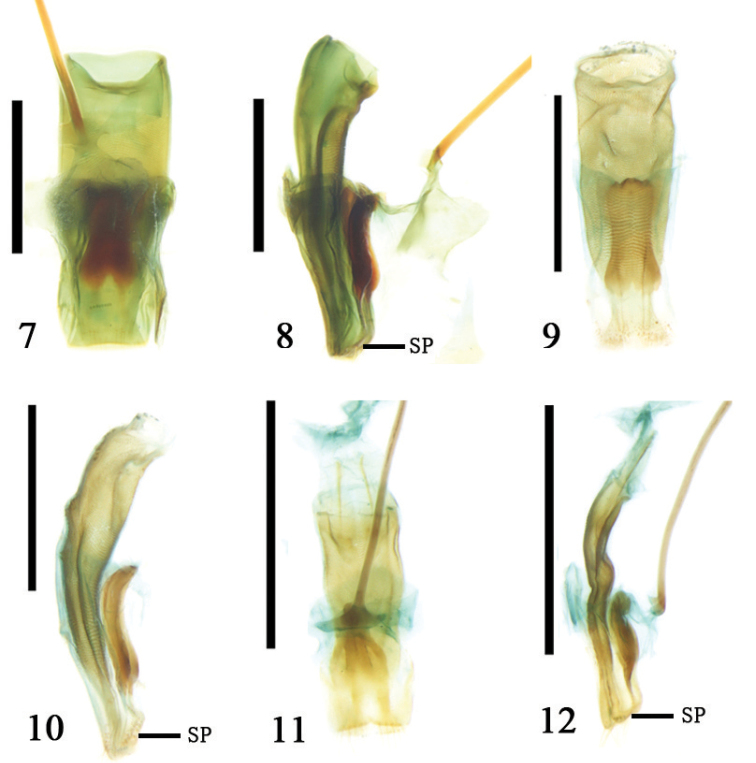
**7–8.** 8^th^ abdominal segment of *Temnaspis nankinea* (Pic). **7** ventral view **8** lateral view **9–10** Part of 8^th^ abdominal segment of *Poecilomorpha discolineata* (Pic) **9** ventral view **10** lateral view **10–12** 8^th^ abdominal segment of Zeugophora (Pedrillia) bicolor Kraatz **11** ventral view **12** lateral view; Abbreviations: sclerotized plate (SP); scale line = 1.0 mm.

The 8^th^ and 9^th^ abdominal segments (Figs 1, 2) of females in the Megalopodinae are hidden within the abdominal cavity. These are covered by the large pygidium (the tergite of the 7^th^ abdominal segment). The 8^th^ abdominal segment (Fig. 2) is long. Most of the surface of tergite 8 is membranous and slightly sclerotized. Sternite 8 (the genital plate) is shorter than tergite 8. It is an X-shaped, strongly sclerotized apodeme with an irregular surface (Fig. 6). Its middle is partly depressed with a convex lateral side. The anteromedian margin is distinctly prominent (Figs 5–10). The spiculum gastrale is thin, long, and slightly curved (Figs 1, 4), with a slightly thickened tip. The anterior portion reaches to the 1^st^ abdominal cavity or is found slightly beyond the apical portion of the first abdominal segment. Along the apical median of the spiculum gastrale, there is a small groove attached to the longitudinal muscle. The base of the spiculum gastrale is connected to sternite 8 by a loose, slightly sclerotized membrane connecting it with the base of the spiculum gastrale (Figs 2, 4, 7, 8).

The lateral side of the tergite and sternite is connected tightly by a membrane that extends from the apex of the abdominal segment to form a flattened tube that is slightly curved on its ventral side (Figs 8, 10). This apical flattened tube extends inward to the body cavity to form a loosely membranous genital pocket. The membrane of the genital pocket is thick and has many sclerotized rings (Fig. 10). Its anterior portion is broad but the posterior portion is narrow (Figs 9–10), the anterior is located in the 4^th^ abdominal segment cavity. The anterior opening of the flattened tube is mainly occupied by the bursa copulatrix, median oviduct, and the apical rectum. The posterior (i.e., the apex of the flattened tube) is the opening of the genital pocket and vagina to the outside. Apical margins of tergite 8 and sternite 8 rarely contained a row of setae (Figs 7–12). The margin of sternite 8 is slightly shorter than that of tergite 8. There is a transverse, oblique, sclerotized plate, which is connected with the apical margin of sternite 8. This plate usually covers the opening of the genital pocket (Figs 5, 8, 10, 12).

**Figures 13–17.**
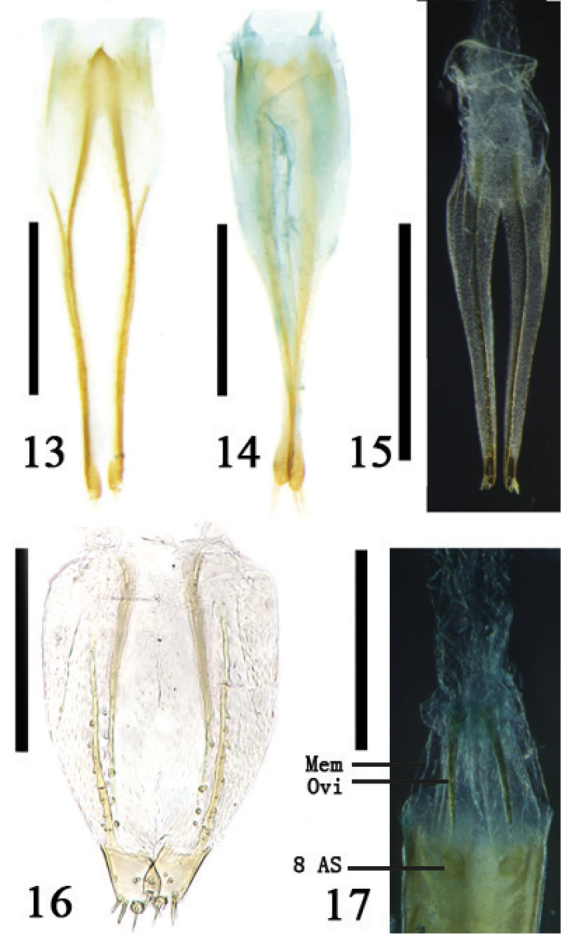
**13–16.** Ovipositor **13** *Temnaspis fraxini* (Komiya), ventral view **14** *Poecilomorpha cyanipennis* (Kraatz), ventral view **15** Zeugophora (Pedrillia) dimorpha (Gressitt), ventral view **16** *Zeugophora cyanea* Chen, ventral view **17** the connecting of ovipositor and 8^th^ abdominal segment, Zeugophora (Pedrillia) bicolor Kraatz, ventral view; Abbreviations: membrane (Mem); ovipositor (Ovi); 8^th^ abdominal segment (8 AS); scale line = 0.5 mm.

The 9^th^ abdominal segment is long and all of its components are wrapped in a genital pocket. The ovipositor is a long scissor-like, strongly sclerotized, basal half portion that is broad and flattened. Its two oblique palpi are separated on the posterior portion. Their apices are generally close to each other. The outer margin of the ovipositor is slightly flattened (Figs 13–16). A majority of the components associated with the ovipositor fuse but do not have a clear boundary, lacking a proctiger and median plate (Figs 13–16). The paraproct and coxite are fused to form a flattened sclerite at the base. The base of the ovipositor is connected by a membrane to the genital pocket (Fig. 17). The coxite is rather long, with sensory setae on the inner side. The apex of the coxite is cylindrical. The stylus is rather small and connected to the outer margin of the coxite. There are rather long sensory setae on the tip of the stylus. The valvifer is fused with the coxite (Figs 13, 15, 16). The ovipositor has one or two pairs of baculi and one pair extends from the base of the ovipositor backward to the coxite. The bases of the two baculi are attached or alternatively can be found proximal to each other. The ovipositor has one or two pairs of baculi. One pair extends from the base of the ovipositor backward to the coxite; the bases of these two baculi are connected or close to each other. Another pair (if there are two pairs) of baculi are rather short and extend from the coxite to the middle of the ovipositor, the apex of the baculus is free or fused to that of the first pair on each side (Figs 13, 15, 16).

The main muscles of the 8^th^ abdominal segment in *Temnaspis nankinea* (Pic) were examined. It was found that, on the genital plate, there is a large paired bundle of fan-shaped muscles between the median and the lateral apodeme of the anterior margin of sternite 8 (Figs 2, 3, 5), which arises from the lateral side of the longitudinal section of the pygidium. The muscles of the spiculum gastrale are comprised of longitudinal muscles and transverse muscles. The longitudinal muscles consist of left and right bundled and rather large muscles (Fig. 4), which extend inclining backward and connect to the dorsal side of the bursa copulatrix. On the lateral tendon of the basal spiculum gastrale, there are two short bands of transversal muscles (Fig. 4), the other end of which connects to the basal lateral angle of the pygidium. The ventral side of the bursa copulatrix has a pair of longitudinal muscle fibers along the inside of the genital plate, extends to the apical margin of sternite 8, and connects to the lateral margin. The egg passes through the median oviduct to the genital chamber and can be discharged through the vulva. The genital plate is located on the ventral side of the genital pocket. In the inner part of the genital plate, there is a concave area which perhaps guides the ovipositor palpi and ovulation.

The structure and morphology of female genitalia of the subfamilies Zeugophorinae and Megalopodinae are very similar. However, their body sizes and external morphologies differ significantly. The genital pocket is approximately cylindrical, its anterior end is narrow, and its posterior portion is flat and generally broadly extended beyond the genital plate. Apical margins of tergite 8 and sternite 8 are nearly equal in length, and rarely contained a row of setae. The ventral apical margin is slightly sclerotized, and the opening of the genital pocket does not close tightly. The curved portion of the flattened tube is located far from the posterior opening. The coxite generally does not exceed the curved portion. The shape of the genital plate is different from that of the Megalopodinae. The genital plate in the Zeugophorinae is cordate in some species (Figs 11, 12), but nearly X-shaped in other species, similar to those in Megalopodinae, such as *Zeugophora scutellaris* Suffrian, Zeugophora (Pedrillia) yunnanica Chen and Pu, Zeugophora (Pedrillia) dimorpha (Gressitt), etc.

### Cerambycidae

**Figures 18–21.**
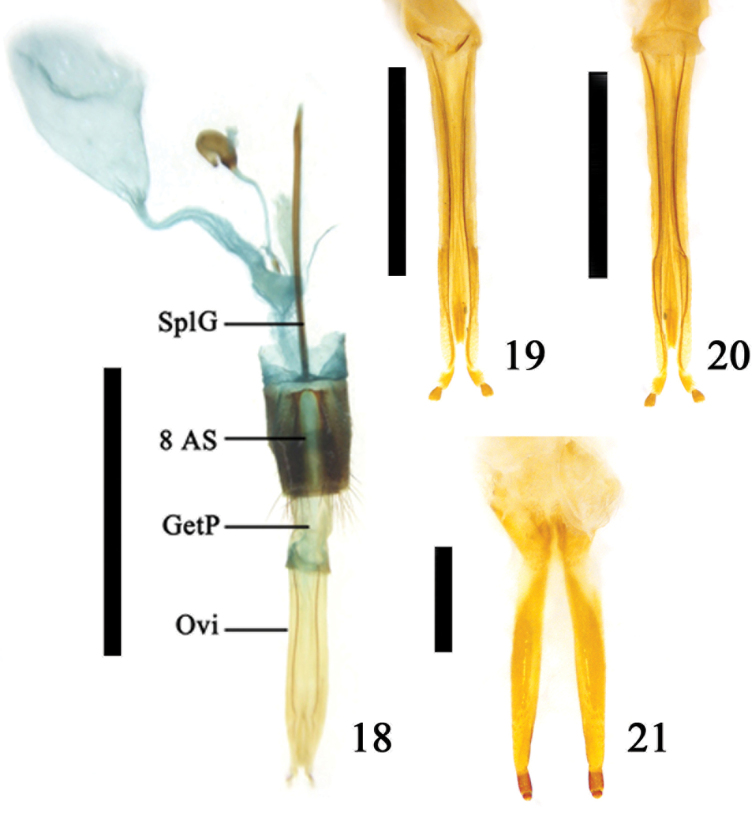
**18** Part of internal reproductive system and ovipositor of *Aromia bungii* (Faldermann), ventral view **19–20** ovipositor of *Leptura annularis annularis* Fabricius **19** dorsal view **20** ventral view **21** ovipositor of *Glenea pulchra* Aurivillius. Abbreviations: genital pocket (GetP); ovipositor (Ovi); spiculum gastrale (SplG); 8^th^ abdominal segment (8 AS); scale line = 1.0 mm (Figs 19–20); scale line = 5.0 mm (Figs 18, 21).

In the majority of members of the family Cerambycidae the tergite and sternite of the 8^th^ abdominal segment are combined to form a flattened segment (Fig. 18). The tergite and sternite are generally equal in length and the apical margin generally contains setae along each side, or alternatively can be found on the lateral angle near the apical margin of the setae plexus (Fig. 18). Inside the apical margin of the 8^th^ abdominal segment there is an inward fold, which extends to the body cavity to form a genital pocket (Fig. 18). The apical opening of the 8^th^ sternite is the posterior opening of the genital pocket, which generally is not closed tightly. Although the lateroapical margin near the posterior opening is slightly dark or slightly sclerotized it is in fact not a sclerite. The genital pocket is stiff and membranous, and its surface has many sclerotized rings. The thickness of the membrane differs among different species. It generally stretches from the apex of the 8^th^ abdominal segment to the basal ovipositor and connects with the ovipositor by a membrane. Morphological variation of the 8^th^ abdominal segment is extremely variable in different species of cerambycids (Figs 18, 20, 23, 24).

**Figures 22–27.**
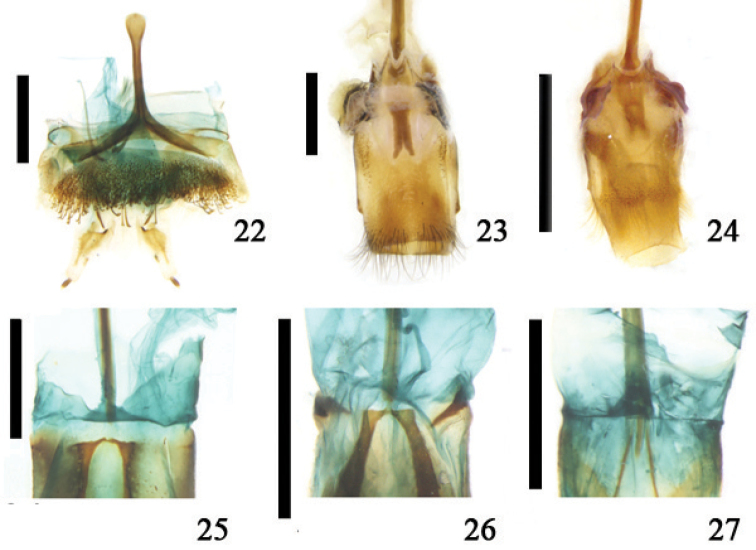
Part of 8^th^ abdominal sternite, ventral view **22** *Anoplistes halodendri* (Pallas) **23** *Glenea centroguttata* Fairmaire **24** *Thyestilla gebleri* (Faldermann) **25** *Aromia bungii* (Faldermann) **26** *Leptura annularis annularis* Fabricius **27** *Asemum amurense* Kraatz; scale line = 1.0 mm.

In general, the genital pocket is cylindrical in shape, but variable among different species. The variation of genital pocket is correlated with the change of the 8^th^ abdominal segment. The spiculum gastrale is thin and long, almost four times the length of sternite 8 and it also has a rod-like or slightly thick apex (Fig. 18). In some species the spiculum gastrale, which is tightly connected to sternite 8 by a tendon, moves freely (Figs 23–26), but a few species lack this joint between the spiculum gastrale and sternite 8 and do not move freely (Figs 22, 27). The species that have this joint are listed as follows (Figs 23–26): *Aegosoma sinica sinica* (White) (Prioninae), *Gaurotes virginea aemula* (Mannerheim), *Leptura annularis annularis* Fabricius (Lepturinae), *Aromia bungii* (Faldermann) (Cerambycinae), *Monochamus alternates* (Hope), *Bacchisa comata* (Gahan), *Oberea formosana* Pic, *Glenea centroguttata* Fairmaire, *Phytoecia rufiventris* Gautier des Cottes, *Paraglenea fortunei* (Saunders), *Thyestilla gebleri* (Faldermann), *Eodorcadion brandti* (Gebler) (Lamiinae); The species that have no joint are as follows: *Asemum amurense* Kraatz (Spondylidinae), *Anoplistes halodendri* (Pallas) (Figs 22, 27), *Purpuricenus temminckii* (Guérin-Méneville) (Cerambycinae). No species were lacking a spiculum gastrale in the Cerambycidae.

The 9^th^ abdominal segment is generally elongated and membranous (Fig. 18). In a stationary state, all components of the 9^th^ abdominal segment are located within the genital pocket. The ovipositor is also elongated and gradually narrows down to a posterior portion (Figs 18–21). Two palpi are usually located near the apex. The components of the ovipositor are generally complete, with all sections containing baculi (Figs 19–20). The proctiger is rather short or absent and the pair of baculi are located on each side (Fig. 19) (only the proctiger in the subfamily Philinae is elongated, Saito 1993). The paraproct is generally elongated, and on its basolateral side its baculus is long and straight. The valvifer is generally fused with the coxite. The apex of the coxite is cylindrical and its outer part is generally swollen (Figs 19–20). It is generally longer than the baculi. The stylus is located on the tip or lateroapical margin of the coxite (Figs 19–21). The ventral side of the ovipositor generally has a median plate (Figs 19–20). In some species of Cerambycidae, the ovipositor is reduced, but can be recognized from the site of the reserved baculus. They generally contain a pair of long dorsal baculi on the dorsal side of the ovipositor in the Cerambycidae (except in the Lamiinae, Saito 1993) (Fig. 21). The dorsal baculi extends from the posterior of the coxite to the base of the ovipositor.

**1. Prioninae**

The 8^th^ abdominal segment in the subfamily Prioninae (genus *Aegosoma*) is exposed to the outside with the posterior extending into a cone-like shape. The genital pocket folds inward from the apex of the 8^th^ abdominal segment. The apical portion of the genital pocket is narrow and its membrane is rather thick and tightly wrapped in the center forming a thin pipe. The apex of the spiculum gastrale is slightly thickened and is connected to the sternite by a joint. The components of the ovipositor are sectioned clearly with an outward apex. The coxite is slightly swollen. The stylus is small and located on the lateral side of the coxite.

**2. Spondylidinae**

The tergite of the 8^th^ abdominal segment in the subfamily Spondylidinae (genus *Spondylis*) is sclerotized and the ventrolateral side of the tergite is folded. Most portions of the sternite are membranous and a sclerite is found on either side. The apical margin of the 8^th^ abdominal segment is folded inward to form a genital pocket. The spiculum gastrale is located between the sclerites of the sternite centroapical margin, which connects to the sternite without a joint. The components of the ovipositor are complete and easily viewed. The coxite is slightly swollen. The stylus is located on the lateral side of the coxite.

**3. Cerambycinae**

In the genus *Aromia*, the length of the 8^th^ abdominal segment is longer than its width (Fig. 18). The tergite is sclerotized in its central portion and is membranous with the lateral margins folding towards the ventral side. Most portions of the sternite are slightly sclerotized. The apical margin of the 8^th^ abdominal segment folds inward to form a genital pocket. There is a joint between the spiculum gastrale and the sternite. The components of the ovipositor are distinct with the stylus located on the apex of the coxite (Fig. 18). In the genera *Anoplistes* and *Purpuricenus*, the 8^th^ abdominal segment is short, broad, and flattened. The anterior margin of the sternite is slightly sclerotized and the setae cluster contains a mixture of long and short setae. The lateral side of the tergite is slightly sclerotized. Species of both genera *Anoplistes* and *Purpuricenus* do not have genital pockets (Fig. 22). Their spiculum gastrale is short. There is no joint between the spiculum gastrale and the sternite. The ovipositor is short and lacks a proctiger. The ovipositors of the latter two genera are membranous. The remaining components of the ovipositor show clear boundaries. The coxite is swollen and its lateral side has setae. The stylus is located on the lateral side of the coxite.

**4. Lepturinae**

In the genus *Leptura*, the length of the 8^th^ abdominal segment is longer than its width and its tergite is connected to the sternite via a membrane that forms a flattened segment. The tergite and the sternite are slightly sclerotized and the apical margin of the 8^th^ abdominal segment folds inward to form a genital pocket. There is a joint between the spiculum gastrale and the sternite. The ovipositor is rather long, its posterior portion extends outward, making the components of the ovipositor clearly visible. The stylus is located on the apex of the swollen coxite (Figs 19–20).

**5. Lamiinae**

Lamiinae is a subfamily that includes a variety of taxa. We observed the genitalia of the following genera: *Monochamus*, *Oberea*, *Glenea* (Fig. 21), *Bacchisa*, *Paraglenea*, *Phytoecia* and *Thyestilla*. The tergite and sternite of the 8^th^ abdominal segment is generally sclerotized and connect to form a flattened segment. The tergite and sternite are usually equal in length. The posterior opening of the 8^th^ abdominal segment generally does not close tightly. Mostly, on the apical margin of the 8^th^ abdominal segment, there are setae and a seta cluster is located at the near corners. The color of these setae is generally dark or slightly sclerotized but they do not form a sclerite. In other genera such as *Thyestilla*, *Phytoecia*, and *Paraglenea*, the apex of the 8^th^ abdominal segment is membranous, forming the apex of the genital pocket. It is similar to the apex found in females of the Megalopodidae but their tergite and sternite margins are sub-equal in length and not tightly closed. The spiculum gastrale is thin, long and is connected to the sternite with a joint. We found that the apex of the spiculum gastrale in the genus *Eodorcadion* is not regular in shape and has an angled apodeme at its apex for muscle attachment. The 9^th^ abdominal segment is long. The ovipositor is narrow from its base to apex. The apex of the ovipositor is not inclined towards the outer part. The coxite is not swollen and does not contract at its base. The outer part of the coxite is comparatively straight (Fig. 21). Most of the components of the ovipositor are present. The proctiger is rather short or absent. The paraproct is shorter than in other taxa in the Cerambycidae but the structures are visible. The valvifer is generally fused to the coxite. The stylus is small and generally located on the apex of the coxite.

Morphological variation of female genitalia in the Cerambycidae is rather minimal compared to the Chrysomelidae. Females in the Cerambycidae usually lay their eggs in tree bark cracks, under tree bark or in soil (Saito 1993). We find that the genitalia of *Anoplistes halodendri* (Pallas) (Cerambycinae) and *Purpuricenus temminckii* (Guérin-Méneville) (Cerambycinae), are very different from those found in more common species of Cerambycidae but are closer to the chrysomelids. Their 8^th^ abdominal segment does not have a genital pocket. Their spiculum gastrale is short and is connected to the sternite without a joint. Their ovipositors are rather short. In addition, two species *Phytoecia rufiventris* Gautier des Cottes, *Thyestilla gebleri* (Faldermann) consume herbs, and their life history and morphology require further investigation. We found that the apex of their abdominal segments were membranous differing from the Cerambycidae.

### Orsodacnidae

**Figures 28–30.**
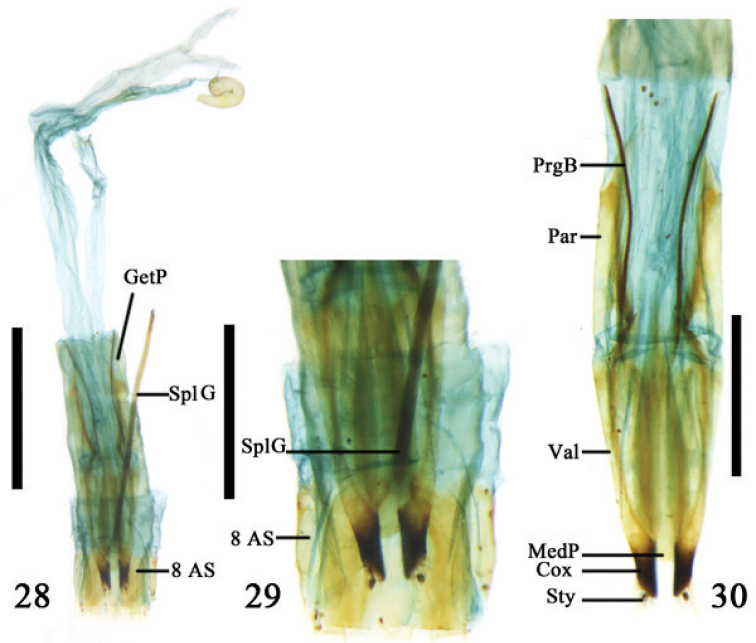
Ovipositor and 8^th^ abdominal segment of *Orsodacne cerasi* (Linnaeus) **28–29** ventral view **30** dorsal view; Abbreviations: coxite (Cox); genital pocket (GetP); median plate (MedP); paraproct (Par); baculus of proctiger (PrgB); spiculum gastrale (SplG); stylus (Sty); valvifer (Val); 8^th^ abdominal segment (8 AS); scale line =0.5mm.

Abdominal segment 8 is sub-quadrate. The tergite and sternite are weakly sclerotized and are connected via a membrane on lateral sides forming a flattened cylindrical segment. The central portion of the tergite is membranous while the central portion of the sternite has one weakly sclerotized sclerite without an apodeme (Fig. 28). The spiculum gastrale is long and thin, connecting with sternite 8 via a ligament (Fig. 29). The inside of sternite 8 has a membranous genital pocket that is as thin as a common inter-segmental membrane, and covers the outside of the 9^th^ abdominal segment. The 9^th^ abdominal segment is long and slightly flattened. The components of the ovipositor are sclerotized with clear boundaries. The proctiger is rather long and membranous but slightly shorter than the ovipositor. The ovipositor has a pair of baculi on its lateral side. The proctiger connects with the paraproct at its base. The paraproct is long and narrow and it has one long baculus whose anterior connects with the baculus of the valvifer. The coxite is long with a cylindrical apex and is strongly sclerotized. The coxites are parallel in the central part and the apices do not reach outward. The apical margin is surrounded by long setae and its tip is oblique and truncated. The stylus is rather thin but not long and does not protrude. Its apex has long setae (Fig. 30). The valvifer is long and narrow but can be distinguished from the coxite. The base of the valvifer connects with the paraproct. The median plate is located on the ventrocentral of the valvifer. The structure of the external genitalia in this family is rather primitive for Chrysomeloidea.

### Chrysomelidae

In this family, the inside of the 8^th^ abdominal segment has no genital pocket. Sternite 8 is usually reduced and the spiculum gastrale has no joint. The length of the 9^th^ abdominal segment is usually shorter than the width of its base, and there is less membrane compared to the Megalopodidae, Cerambycidae, and Orsodacnidae. Morphological of the ovipositor is variable and has all components although some portions are reduced or fused. The baculi of the ovipositor are poorly developed. This includes the early branched groups (the Sagrinae and the Eumolpinae) and the late appeared group Alticinae and Cassidinae.

**1. Sagrinae**

**Figures 31–34.**
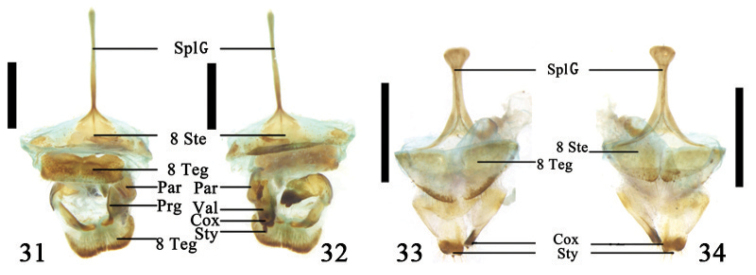
Ovipositor and 8^th^ abdominal segment **31–32** Sagra (Sagrinola) mouhoti Baly **31** dorsal view **32** ventral view **33–34** *Lilioceris cheni* Gressitt & Kimoto **33** dorsal view **34** ventral view; Abbreviations: coxite (Cox); paraproct (Par); proctiger (Prg); spiculum gastrale (SplG); stylus (Sty); sternite 8 (8 Ste); tergite 8 (8 Teg); valvifer (Val); Scale line = 1.0 mm.

The genus *Sagra* has a well-developed sternite 8 and a moderate to long spiculum gastrale. The apex of the spiculum gastrale is slightly expanded and its ovipositor is short and thick. The proctiger is membranous with a sclerite on each side of the proctiger. The apex of the proctiger inserts into tergite 8. The paraproct is strongly sclerotized and thick, while its apex is connected to the valvifer, coxite, and stylus. There is a median plate between the valvifer on its ventral side. This is representative of primitive types in the Chrysomelidae (Figs 31–32).

**2. Bruchinae**

The 8^th^ abdominal segment, associated with members of the genus *Callosobruchus*, is quadrate. The ventral side of the tergite is close to the proctiger. The central part of the tergite is membranous and its lateral side is sclerotized. The sternite is membranous. The tergite and sternite are connected to each other on the lateral side. The spiculum gastrale is short, about 1.5 times the length of the sternite, and lacks a broad apex. In *Callosobruchus chinensis* (Linnaeus) the spiculum gastrale extends forward turning backward at its base. The length of the 9^th^ abdominal segment is almost equal to the length of the 8^th^ abdominal segment. The proctiger is approximately triangular and membranous with sclerotized lateral parts. The base of the proctiger is connected to the paraproct. The ovipositor is small. The valvifer, coxite, and stylus lack clear boundaries.

**3. Criocerinae**

This subfamily is similar to the Sagrinae but the spiculum gastrale is relatively short (Figs 33–34). The components of the ovipositor show clear boundaries but the valvifer generally fuses with the coxite or the paraproct. In some cases the stylus may be either indistinct (genus *Oulema*) or distinct according to species (e.g. *Lilioceris* and *Lema*). The components of the ovipositor have clear boundaries in the genus *Mecoprosopus* although their valvifer is fused with the paraproct.

**4. Donaciinae**

**Figures 35–39.**
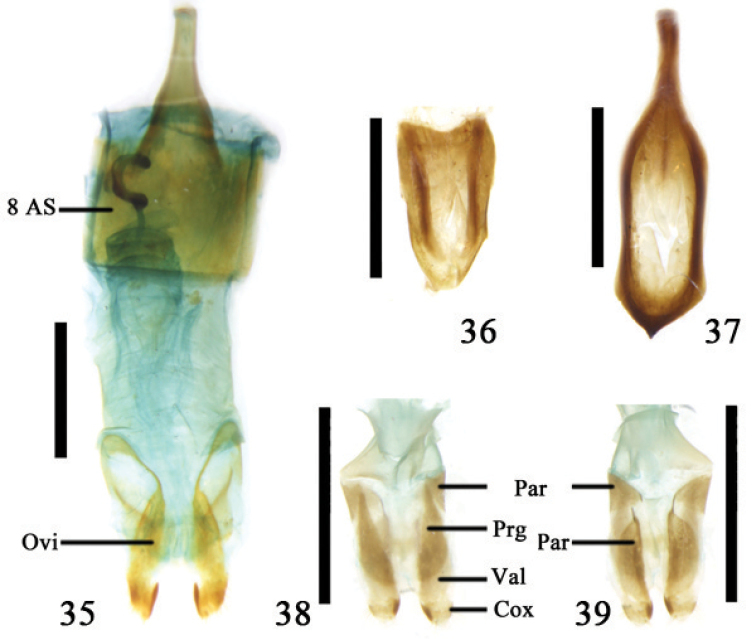
**35** Ovipositor and 8^th^ abdominal segment of *Donacia longicornis* Jacoby **36–37** *Plateumaris weisei* (Duvivier) **36** tergite 8 **37** sternite 8 **38–39** ovipositor of *Macroplea mutica* (Fabricius) **38** dorsal view **39** ventral view; Abbreviations: coxite (Cox); ovipositor (Ovi); paraproct (Par); proctiger (Prg); valvifer (Val); 8^th^ abdominal segment (8 AS); scale line = 1.0 mm.

Donaciinae is an aquatic subfamily. The 8^th^ and 9^th^ abdominal segments are slightly sclerotized. The spiculum gastrale of the 8^th^ abdominal segment is rather long. The 9^th^ abdominal segment is significantly long and membranous. The proctiger is usually long, triangular and its basolateral side is connected to the paraproct. The valvifer usually fuses with the coxite. The stylus is generally indistinct. The area of sensory setae is located on the surface of the tip of the coxite and may be interpreted as a stylus (Figs 35, 38, 39). It is only in the genus *Macroplea* that the ovipositor has all its components with clear boundaries. The female of *Donacia provostii* Fairmaire inserts her ovipositor in a small hole on the dorsal surface of a leaf and lays eggs on the leaf’s ventral side. The inter-segmental membrane between the 8^th^ and 9^th^ abdominal segments is very long. In the genus *Plateumaris* the 8^th^ abdominal segment is sclerotized and specialized forming a flattened sheath (Figs 35–36) with the ovipositor in this sheath. Members of this genus have no proctiger. We found that the inter-segmental membrane between the 8^th^ and 9^th^ abdominal segments in *Plateumaris socia* (Chen) was rather long.

**Figures 40–42.**
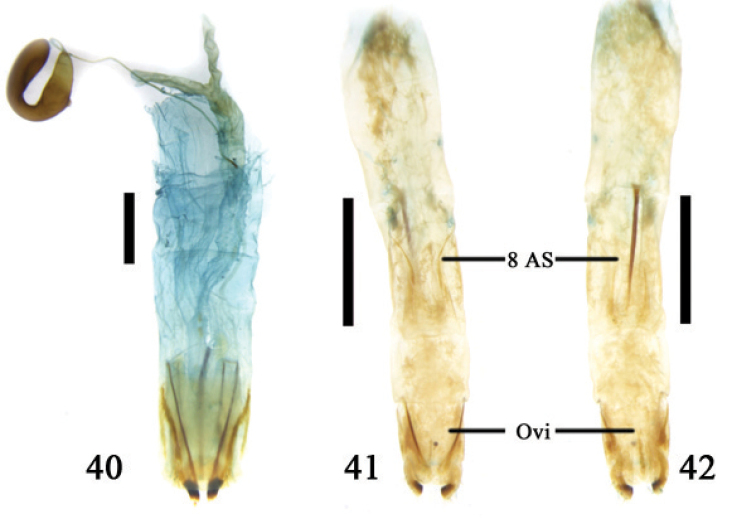
**40** ovipositor and 8^th^ abdominal segment of *Platycorynus* sp. **41–42** ovipositor and 8^th^ abdominal segment of *Colasposoma dauricum dauricum* Mannerheim; Abbreviations: ovipositor (Ovi); 8^th^ abdominal segment (8 AS); scale line = 1.0 mm.

**5. Eumolpinae**

**Figures 43–45.**
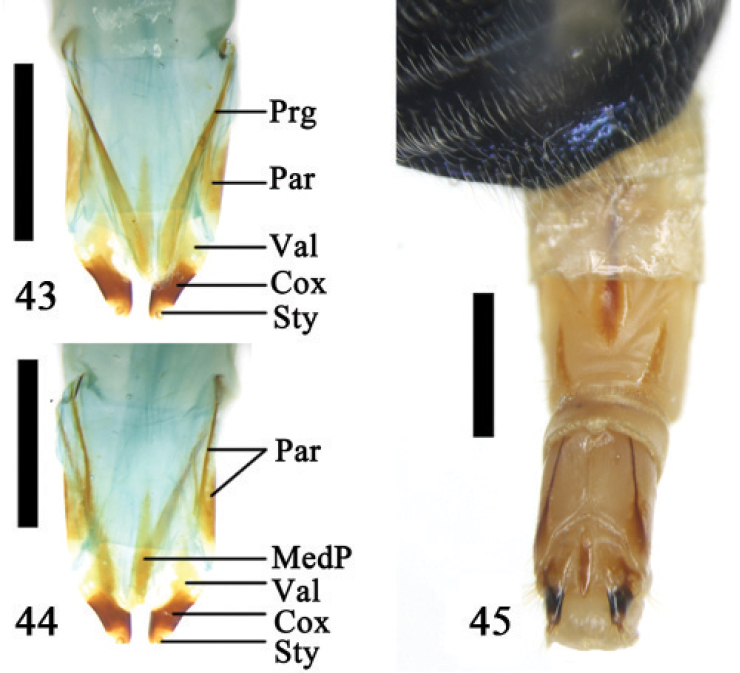
*Chrysochus chinensis* Baly **43–44** ovipositor **43** dorsal view **44** ventral view **45** ovipositor and 8^th^ abdominal segment, ventral view; Abbreviations: coxite (Cox); median plate (MedP); paraproct (Par); proctiger (Prg); stylus (Sty); valvifer (Val); scale line = 1.0 mm.

**Figures 46–47.**
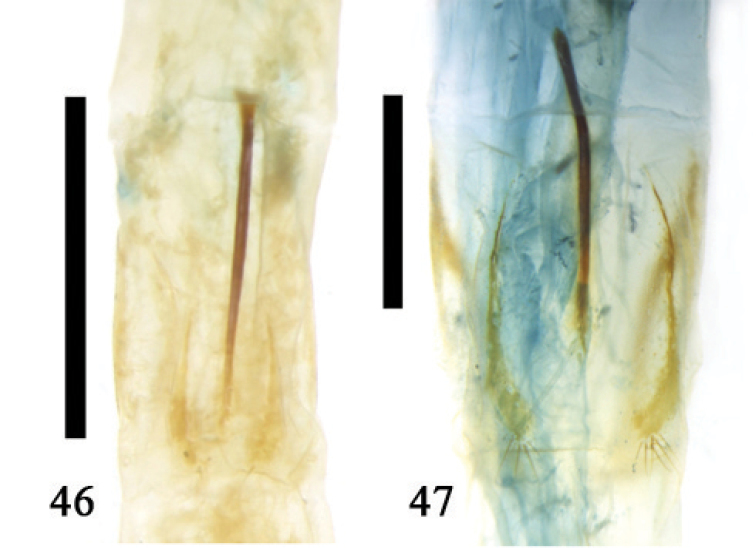
8^th^ abdominal sternite, ventral **46** *Colasposoma dauricum dauricum* Mannerheim **47** *Platycorynus* sp.; scale line = 1.0 mm.

The reproductive segment of this subfamily is distinctive in the Chrysomelidae. In the genera *Platycornus*, *Colasposoma* and *Chrysochus* the tergite and sternite of the 8^th^ abdominal segments fuse laterally, forming a flattened cylinder. The lateral side of tergite 8 has a sclerotized area and sternite 8 is sclerotized (Figs 46–47) but the sclerite of sternite 8 is longer than the length of tergite 8. The inter-segmental membrane of the 8^th^ and 9^th^ abdominal segment is very long forming a membrane surrounding the outside of the 9^th^ segment (Figs 40–42). The 9^th^ abdominal segment is longer than broad at its base and its membrane is developed. The ovipositor has all components with clear boundaries and it has developed baculi. The proctiger is membranous, long, triangular, and its apical margin is emarginated with its lateral side connected to the paraproct (Fig. 43). The ventrocentral portion of the ovipositor has a long and narrow median plate (Fig. 44). We dissected one specimen of *Chrysochus chinensis* Baly. Its ovipositor was much elongated and 1/2 the length of the body (from the head to the apex of the elytra) (Fig. 45).

**6. Lamprosomatinae**

In the genus *Oomorphoides*, the lateral side of tergite 8 is sclerotized and sternite 8 is slightly sclerotized without a spiculum gastrale (Fig. 53). The middle of its proctiger is membranous with sclerotized lateral sides. The paraproct is fully developed connecting to the proctiger on its lateral side. The ovipositor has all components showing clear boundaries. The stylus is located on the tip of the coxite, which is fused with the valvifer at its base. The ventrocentral portion of the ovipositor has a median plate.

**7. Cryptocephalinae**

**7.1. Cryptocephalini**

In the genus *Cryptocephalus* the proctiger is membranous and the central portion of its apex is emarginated with the lateral side sclerotized with sharp protrusions. The paraproct is developed with most of this structure connected to the proctiger. The ventral portion of the paraproct is located on the lateral side of the 9^th^ segment. The coxite is a wedge-shaped sclerite, extending from central region to the outside, which connects it with the valvifer. This valvifer connects to the paraproct. The inner side of the coxite protrudes backward while the outer part is an inclined plate with some setae. This inclined plate should be the stylus (Figs 51–53).

**7.2. Clytrini**

In genus *Aspidolopha*, the ovipositor has all components but the stylus is tiny. The coxite and valvifer have clear boundaries while the paraproct is strongly sclerotized.

**7.3. Fulcidacini**

In the genus *Chlamisus*, sternite 8 is membranous and the vaginal palpi are robust with no stylus. Most portions of the boundaries between the valvifer and coxite are invisible, with just a small piece located inside being visible. The valvifer is very long.

**8. Synetinae**

**Figures 48–53.**
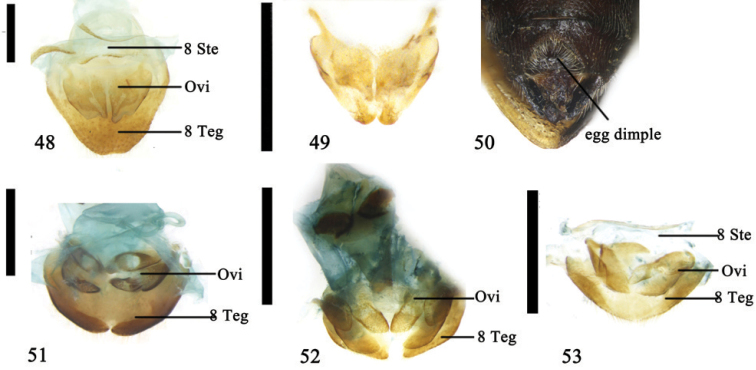
**48–50** *Syneta adamsi* Baly **48** 8^th^ abdominal segment, ventral view **49** ovipositor, dorsal view **50** egg dimple of last abdominal sternite, ventral view **51–53** Ovipositor, ventral view **51** *Physosmaragdina nigrifrons* (Hope) **52** species of Cryptocephalinae **53** *Oomorphoides yaosanicus* (Chen); Abbreviations: ovipositor (Ovi); sternite 8 (8 Ste); tergite 8 (8 Teg); scale line = 0.5 mm (Figs 48, 49, 51, 53); scale line = 1.0 mm (Figs 52).

The last visible tergite (pygidium) extends backward (Fig. 48). Sternite 8 is membranous but its margin is sclerotized without a spiculum gastrale (Fig. 48). Segment 9 is broader than its length. The proctiger and apex of the ovipositor palpi are on the ventral side of the pygidium. The proctiger is a broad plate and weakly sclerotized with an emarginated and sclerotized apical margin. The paraproct is broad with its lateral margin closely connected with the proctiger. The coxite is sub-triangular instead of cylindrical. Because of this variation in the coxite there is no obvious or prominent stylus. However on the inclined plane of the outside of the coxite there is a long membranous surface that may be the stylus according to the structure of the ovipositor. The valvifer is located on the base of the coxite, with the outer part of the valvifer connected to the paraproct (Fig. 48). There is a smooth inner wall with the egg dimple on the apical margin of the 7^th^ abdominal segment (Fig. 50).

**9. Chrysomelinae**

**Figures 54–59.**
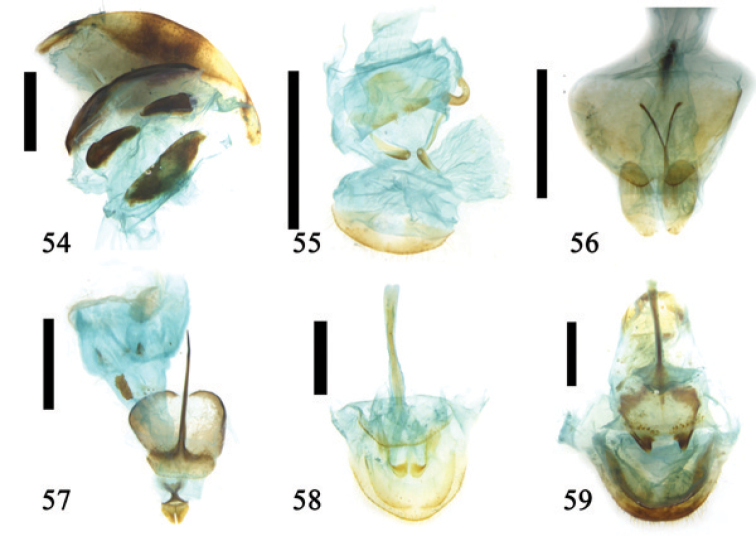
Ovipositor and 8^th^ abdominal segment **54** *Agasta formosa* Hope **55** *Chrysomela populi* Linnaeus **56** *Paleosephraria* **57** *Mimastra limbata* Baly **58** *Podontia affinis* (Gröndal) **59** *Podontia dalmani* Baly; scale line = 1.0 mm.

The central portion of tergite 8 is membranous, and it is sclerotized laterally. The sternite is normal or reduced. The whole subfamily, except in the genus *Timarcha*, has no spiculum gastrale. The central section of proctiger is membranous, sometimes reduced. The basolateral margin of the proctiger is connected to the paraproct. The ovipositor is short, robust, and palp-like, its components are fused and it may have a median plate. Sternite 8 in the genus *Chrysomela* is membranous. Its apical margin is sclerotized and centrally emarginated while the ovipositor is rod-like, weakly sclerotized with a rather thin base. The components of the ovipositor are fully fused together, with the stylus circle-shaped at the apex (Fig. 55). In the genus *Agasta*, the ovipositor palpi are strongly sclerotized with visible components lacking a clear boundary (Fig. 54). Each side of the proctiger in the genus *Chrysolina* has a strongly sclerotized sclerite. The outer part of the base is connected to the paraproct. The palpi of the ovipositor are strongly sclerotized and the valvifer fuses with the coxite. The sensory setae area at the tip of the coxite should be the stylus. The stylus and coxite in such genera as *Colaphellus* and *Gastrophysa* have clear boundaries. The latter genus has no proctiger and the center of its ventral ovipositor has a median plate.

**10. Galerucinae**

While the morphology of the spiculum gastrale is variable (Figs 56–57) most are long and narrow as in the genera *Cneorane*, *Galeruca*, *Gallerucida*, and *Monolepta*. In contrast, the spiculum gastrale is rather short in the genus *Paleosepharia* and sternite 8 is reduced to a membrane. In the genus *Morphosphaera*, sternite 8 is normal but the spiculum gastrale is absent while the center of the proctiger is membranous and its lateral sides are sclerotized. Its paraproct is generally not well differentiated. The morphology of the ovipositor in this subfamily is also variable. Generally it lacks a median plate. The ovipositor palpi in the genus *Gallerucida* consist of a pair of parallel, thin, long sclerites. The coxite is cylindrical and the stylus projects long setae at its tip. The valvifer is thin and long and there is no boundary between the valvifer and coxite. The base of the sclerite of the ovipositor palpi may be the paraproct. The inter-segmental membrane of the 8^th^ and 9^th^ abdominal segments is very long. In the genus *Morphosphaera* rod-like components fuse with a rather broad median plate. The ovipositor palpi in genus *Oides* fuses to form a strongly sclerotized rhomboid with a narrow and thin base. Its apical portion is narrow and pointed. The ovipositor in genus *Cneorane* is parallel and lacks a distinct stylus but the sensory setae on its tip are clearly visible. The valvifer fuses with the coxite. The basal sub-rounded sclerites of the ovipositor palpi may be made by the paraproct because there is a clear boundary between the paraproct and the coxite.

**11. Alticinae**

There is no obvious difference between the 8^th^ and 9^th^ abdominal segments in the subfamilies Alticinae and Galerucinae. Both subfamilies lack distinct paraproct. The coxite in the genus *Podontia* is cylindrical and has a small stylus (Figs 58–59). The base of the coxite of *Podontia lutea* (Olivier) has a small transverse sclerite. The base projecting the sclerite of *Podontia dalmani* Baly may be the paraproct (Fig. 59). The ovipositor in the genus *Hemipyxis* diverges at its base with a cylindrical coxite. The apex of this coxite lacks a stylus but bears a couple of sensory setae. The valvifer may be fused with the coxite. The sclerite is divided at the base of the ovipositor and may represent the paraproct. The ovipositor in the genus *Altica* is a simple cylinder. Its coxite is connected to a baculus and the stylus is on the tip of the coxite.

**12. Cassidinae**

In this subfamily the pygidium is tergite 8, and in most species, sternite 8 is reduced to an arc-shaped piece. The spiculum gastrale is usually short and broad, but it is missing in a few species. The ovipositor palpi are rather short, with robust and plate-like apices. The coxite, valvifer, and paraproct are generally fused together. The stylus is usually indistinct. The proctiger is membranous, both sides have rectangular sclerites, base connected with the paraproct.

**12.1. Hispini**

The genus *Octodonta* has no spiculum gastrale and its ovipositor palpi are robust and broad, with apices bearing setae. The paraproct is transverse at the base. Sternite 8 in genus *Lasiochila* is arc-shaped with a short broad spiculum gastrale. Its ovipositor palpi are rather long, but are separated from each other at their bases becoming close to each other at their apices. (Figs 63–64).

**Figures 60–65.**
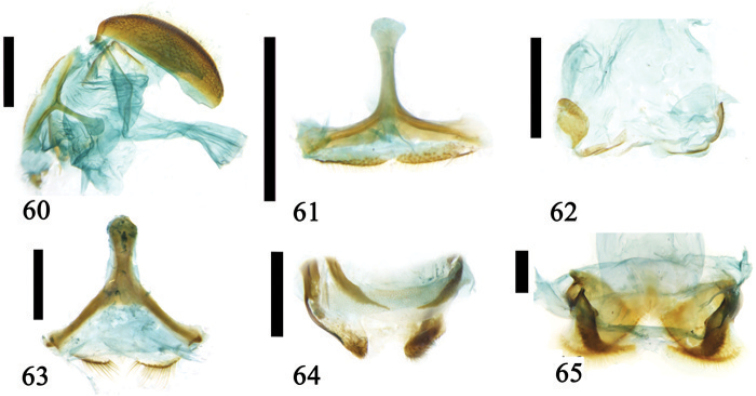
**60** 8^th^ abdominal sternite of *Callispa brettinghami* Baly **61** 8^th^ abdominal sternite of *Callispa nigricollis* Chen & Yu **62** ovipositor of *Callispa nigricollis* Chen & Yu **63** 8^th^ abdominal sternite of *Lasiochila cylindrica* (Hope) **64** ovipositor of *Lasiochila cylindrica* (Hope) **65** ovipositor of *Basiprionota bisignata* (Boheman); scale line = 0.5 mm (Figs 63–65); scale line = 1.0 mm (Figs 60–62).

The central portion of sternite 8 in the genus *Dactylispa* is sclerotized and the spiculum gastrale is rather narrow. Ovipositor palpi are long with distinctly big, thick apices. The stylus is not distinct and the coxite and valvifer fuse. The paraproct is located at the base of the ovipositor.

In the genus *Callispa*, the proctiger is membranous and the anterior margin on the lateral side has a sclerite while the paraproct is reduced to a transverse sclerite at the base of the ovipositor. The ovipositor is plate-like and the stylus, coxite, and valvifer are fused without a clear boundary (Figs 60–62).

**12. 2. Cassidini**

In the genus *Basiprionota*, both proctiger and paraproct structure is similar to that of the genus *Callispa* but the paraproct is located at the base of the ovipositor and is reduced to form an erect sclerite. All parts of the ovipositor fuse into a plate-like structure (Fig. 65).

## Discussion

After examining the external genitalia of females in the Megalopodidae and comparing them to families in Chrysomeloidea, we interpret the following.

**I.** We suggest dividing the female external genitalia of superfamily Chrysomeloidea into cerambycid type and chrysomelid type. In general, adult females with long ovipositors and a spiculum gastrale insert their egg into deeper substrate (plant tissue or soil). In contrast, females with shorter ovipositors and a spiculum gastrale lay eggs on substrate surfaces or shallow sites (Kasap and Crowson 1985). However, in the aquatic genus *Donacia (Cyphogaster)* and in some species in the genus *Colasposoma* (Chrysomeloidea; Figs 40–41), females with short ovipositors or a spiculum gastrale can elongate the inter-segmental membrane and insert eggs into deeper sites. Therefore the formation of a cerambycid type and a chrysomelid type of ovipositor is more reasonable than simply subdividing them into long and short type.

The main characteristics and representative group in these two types are discussed below.

**1. The cerambycid type**

The Megalopodidae, most species of Cerambycidae and the Orsodacnidae belong to this type. The 8^th^ abdominal segment usually develops a genital pocket and the spiculum gastrale is thin and long. Indeed, it is four times longer than the sternite and is connected to the sternite via a joint. The length of its 9^th^ abdominal segment exceeds the width of the base. The ovipositor is generally long with well-developed baculi.

**2. The chrysomelid type**

All chrysomelids, excluding members of the Orsodacnidae and a few species in the Cerambycidae, belong to this type. The 8^th^ abdominal segment has no genital pocket and the spiculum gastrale connects to the sternite without forming a joint. The sternite is up to four times the length of the spiculum gastrale. The length of the distinctive 9^th^ abdominal segment is generally shorter than the width of its base. The components of the ovipositor are relatively short and its baculus is poorly developed or absent.

**II.** Megalopodidae is closely related to subfamily Lamiinae. The morphology of female genitalia in the family Megalopodidae (includes subfamilies Megalopodinae and Zeugophorinae) is significantly different from the chrysomelids. The female genitalia in this family belong to the cerambycid type. The distinctive characteristics are listed as follows: (1) sternite 8 has a strongly sclerotized apodeme, (2) the inner side of the 8^th^ abdominal segment has a stiff genitalia pocket, (3) the spiculum gastrale is connected to the sternite by a joint, (4) the 9^th^ abdominal segment has no proctiger, (5) the paraproct is rather short or absent, (6) the coxite is thin and long, (7) the stylus is much reduced. The family Cerambycidae includes many subfamilies but morphological variation among its subfamilies is rather low. The ovipositor of most species in the Cerambycidae belongs to the cerambycid type. After comparing the morphology of external female genitalia between the Megalopodidae and Cerambycidae we found that the Megalopodidae are most like the subfamily Lamiinae of Cerambycidae. Our research results are congruent with the research of McKenna et al. (2015). It shows that Megalopodidae was included in the clade with Cerambycidae and Orsodacnidae. And it also supports the researcher’s results that Megalopodidae was close to Cerambycidae or within Cerambycidae (Hunt et al 2007, Gómez-Zurita et al. 2007, 2008; Marvaldi et al. 2009). Both Megalopodidae and Cerambycidae have a sternite 8 with strongly sclerotized apodemes. Their ovipositors have no proctiger or are much reduced. Their paraprocts are short or reduced and their coxites are long but their styluses are small. Dutt (1958) described the morphology of female genitalia in *Nupserha bicolor* (Lamiinae). Saito (1993) and Wang (1999) identified the main morphological characteristics of the Lamiinae ovipositor. Those results are similar to our observations of the Megalopodidae. Suzuki (1988, 1994) considered that Zeugophorinae and Megalopodinae derived from the same lamiid-type ancestor, based on the morphology of the internal reproductive systems of both sexes and the hindwing venation. In addition, Saito (1993) found that the females of Lamiinae used their mandibles to bite tree bark or a host plant’s stem to prepare the substrate for oviposition. This habit is unique to all subfamilies of Cerambycidae. Females in the Megalopodidae generally use their mandibles to bite the stems of host plants before laying eggs. In all, these observations about the morphology of female genitalia in the Megalopodidae and Cerambycidae show that both families probably belong to the cerambycid lineage.

**Figure 66.**
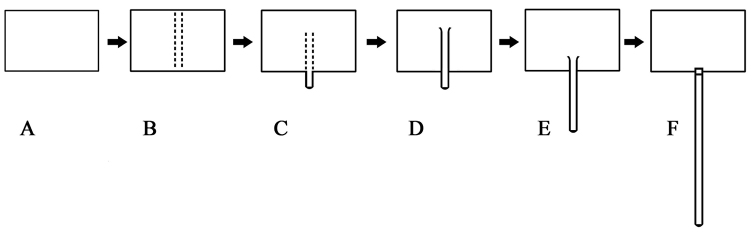
The development of spiculum gastrale **A** sternite 8 **B** sternite 8 central with convex longitudinal ridge **C, D, E** sternite 8 central with convex longitudinal ridge and lateral sides seperated from sternite to form spiculum gastrale without joint **F** spiculum gastrale connected with sternite 8 with joint.

**III.** The evolution of the spiculum gastrale in the Chrysomeloidea. The spiculum gastrale is an apodeme projection extending from the anterior-central region of sternite 8. The muscles attached to the spiculum gastrale correlate the mode of oviposition (see I. Megalopodidae) (Kasap and Crowson 1985). The length of the spiculum gastrale or its absence also correlates with egg laying behavior (Kasap and Crowson 1985). The spiculum gastrale in the Megalopodidae, Cerambycidae, and Orsodacnidae is thin, long, and generally exceeds four times the length of the sternite. It is connected to sternite 8 by a joint at the base. Thus far the spiculum gastrale is present in all three families. In contrast, the spiculum gastrale in the Chrysomelidae is much shorter than in the other families but its length varies from moderate to very short or absent depending on species. The spiculum gastrale is typically absent in such members of the Chrysomelidae as the Lamprosomatinae, Synetinae, and Chrysomelinae (excluding members of genus *Timarcha*). Sternite 8 in the first two taxa is completely reduced while sternite 8 in the latter subfamily has no spiculum gastrale even though sternite 8 is well developed. Sternite 8 in the Eupoda is normal and the development of its spiculum gastrale is also normal and the apical sternite is rather broad. However, in the genus *Sagra* morphological variation is more uniform. The spiculum gastrale in the subfamily Criocerinae is slightly shorter than it is in the genus *Sagra* and in the subfamily Donaciinae. This structure is more distinctive in the genus *Crioceris*. We have not found the sternite without a spiculum gastrale in these taxa. The species in the Donaciinae have a spiculum gastrale slightly shorter than in the genus *Sagra*. The spiculum gastrale in the genus *Sominella* has a hook-like anterior portion. In the genus *Plateumaris* the 8^th^ abdominal segment is a specialized, sclerotized sheath and its apex has vanished. Therefore the spiculum gastrale forms a short and thick handle at the base of the sheath. The spiculum gastrale in subfamily Galerucinae and Alticinae varies considerably with its thin rod shape. It is generally thinner and smaller than in the Eupoda. However, this structure shows greater variation in some genera in the Galerucinae. For example the spiculum gastrale in the genus *Oides* (Galerucinae) is broad, ending in narrow sclerites. Sternite 8 is strongly sclerotized. Species belonging to the genus *Morphosphaera* lack a spiculum gastrale but sternite 8 is well developed. In hispid taxa, most of the spiculum gastrale is broad forming a short plate while most of sternite 8 is reduced to an arc-shaped structure. In contrast, the spiculum gastrale of *Leptispa longipennis* (Gestro) is rather narrow. In the genus *Octodonta* there is no spiculum gastrale, and the development of sternite 8 is normal. In subfamily Eumolpinae, the sternite in some genera (e.g., *Platycornus*, *Colasposoma*, and *Chrysochus*) is rather long while the spiculum gastrale is relatively short or not well developed. In *Colasposoma dauricum* Mannerheim, sternite 8 is centrally convex, with a longitudinal ridge and its lateral side is not completely separated and connects with the sternite. Its apex has no spiculum gastrale (Fig. 45). In genus *Platycorynus*, sternite 8 also has a similar central longitudinal ridge. The anterior portion of this ridge exceeds the anterior of the sternite forming a short spiculum gastrale (Fig. 47). Based on the two above species, we suspect that the spiculum gastrale derives from the longitudinal ridge of the sternite. Based on our morphological analyses of the Chrysomeloidea, we suggest that the spiculum gastrale derived first from the center of sternite 8, then gradually exceeded the original sternite (Fig. 66). The early formation of the spiculum gastrale had no joint, and we regard this as a primary stage. The spiculum gastrale with its joint must have developed relatively late. Our observation is supported by Kasap and Crowson (1985) who also concluded that the spiculum gastrale with its joint was secondary. From an evolutionary point of view, researchers have treated the Cerambycidae as a primary group (Chen 1986, Suzuki 1994), due to the presence of a spiculum gastrale connected to sternite 8 via a joint but this earlier hypothesis is based only on one characteristic.

**IV.** The female genitalia in subfamily Eumolpinae is a typical representative of the primary type. In general, the female genitalia in Orsodacninae, Sagrinae, *Timarcha* (Chrysomelidae) and Eumolpinae are interpreted as primary, because in these taxa female genitalia retain all the basic sclerites: proctiger, paraproct, valvifer, coxite, stylus, median plate (without in *Timarcha*) (Kasap and Crowson 1985). However, we suspect that in the 8^th^ and 9^th^ abdominal segments involved in reproductive behavior the ovipositor is controlled by the muscles of the 8^th^ abdominal segment. Therefore, we believe it is more reasonable to include the 8^th^ and 9^th^ abdominal segment when we try to construct the evolutionary pathway of this taxon, especially when attempting to define ancestral states. We found in some species of Eumolpinae (e.g. *Colasposoma dauricum* Mannerheim) that its spiculum gastrale was not completely separated from sternite 8. This appears more basal compared to well-developed sternites. In addition, most genera in the Eumolpinae (e.g. *Chrysochus*, *Colasposoma*, and *Platycorynus*) with long 9^th^ abdominal segments had ovipositors similar to the cerambycid type. Specifically, their ovipositors have all components, there is a clear boundary, and their long baculi are similar to the Cerambycidae differing from the Chrysomelidae. According to the morphology of the 8^th^ and 9^th^ abdominal segment, it is suggested that the female genitalia of the Eumolpinae represent the ancestral state in the Chrysomelidae.

**V.** Orsodacnidae

This is a controversial group. It was regarded previously as a subfamily in the Chrysomelidae. Mann and Crowson (1981) studied the morphology of adults and larvae in the genus *Orsodacne*; they believed that the ovipositor was very similar to most species in the Cerambycidae. Kuschel and May (1990) made a key to divide families using female genitalia characteristics when they published the new subfamily in the Megalopodidae called the Palophaginae. Their keys show that the Cerambycidae and the Orsodacnidae have common characteristic states (the 9^th^ sternite long, paraprocts with distinct struts or bars and stylus) usually large in female genitalia. Suzuki (1988) thought that some inner reproductive characteristics in the Orsodacnidae were absent in the Chrysomelidae but they were present in some taxa in the Cerambycidae. Our observations on the female genitalia in genus *Orsodacne* are similar to earlier observations by Suzuki (1988). We found that some structures associated with the ovipositor belong to the cerambycid type (including the Megalopodidae and Cerambycidae) and they differ distinctly from the chrysomelid type. The structure of the cerambycid type suggests it represents the ancestral structure. Although we found that the morphology of the reproductive segments in the genus *Orsodacne* differed from those in the Megalopodidae and Cerambycidae, for example sternite 8 has no strongly sclerotized genital plate, the genital pocket is an inter-segmental and long cylindrical membranous membrane, the posterior opening of the 8^th^ abdominal segment has no sclerite plate, and the 9^th^ abdominal segment has a rather long proctiger. These differences are not sufficient to indicate that *Orsodacne* is more closely allied to the chrysomelids.

Crowson (1955) thought that some morphological characteristics of the larvae of *Orsodacne* were similar to the Criocerinae, Donaciinae, *Sagra*, *Clytra*, and Eumolpinae. Cox (1981) also suggested that the Orsodacninae was closer to the Zeugophorinae and Donaciinae based on larva characteristics. There is no doubt that the Orsodacninae are closer to the Galerucinae. In our study, we found that the 9^th^ abdominal segment of *Orsodacne* is very similar to the same structure in some genera (*Colasposoma*, *Platycorynus* and *Chrysochus*) placed in the Eumolpinae. They share the same following features: a long 9^th^ abdominal segment, the ovipositor visibly retains all structures and has developed baculi. However, the 8^th^ abdominal segment in these three genera differs from that in the genus *Orsodacne.* Based on our comparison of the external female genitalia in the Chrysomeloidea and observations by other researchers on the genus *Orsodacne*, we suggest that *Orsodacne* separated from the cerambycid lineage at an earlier period. It moved from bored stems of plants to leaf-eating in the early evolution of the ancestor of Chrysomeloidea, but it keeps some plesiomorphy. It was in the clade that became the earliest independent chrysomelid lineage. Chrysomelidae (include other chrysomelids) was in the clade that evolved later than *Orsodacne*. We agree that the Orsodacnidae was an independent family but a sister family of the Chrysomelidae and it was equal in status to the Megalopodidae and Cerambycidae (Kuschel and May 1990, Reid 1995). Specifically the Chrysomeloidea now includes four families: the Megalopodidae, Cerambycidae, Orsodacnidae, and Chrysomelidae.

**VI.** Synetinae

With a reduced sternite 8, the oviposition behavior in the Synetinae is very similar to the Camptosomata including the subfamily Cryptocephalinae (Cryptocephalini, Clytrini and *Chlamisus*). All of them place a protective layer outside their eggs. They hold each egg by their hind tarsi and keep turning in the egg dimple of the 7^th^ abdominal segment when the egg is expelled from their bodies (Jiang 1983, Yu 1996). Their digestive tracts discharge evenly to cover each egg forming a protective layer and then the egg is kicked out of their bodies (Yu 1996, Jiang 1983). The protective layer is formed by the Kotpresse (German language, meaning fecal press), which is a special structure on the rectum (Lécaillon 1899). The Kotpresse was reported in the Camptosomata (Erber 1968) and Synetinae (Reid 1995). We also found some vertical arrangements of a special Kotpresse on the rectum of *Syneta adamsi* Baly.

The female genitalia of the Synetinae are different from the more common Chrysomelidae. The tergite 8 (pygidium) was exposed and backward, so one could see the exposed apices of the ovipositor from its ventral side. The coxite is triangular lacking a distinct stylus. But on the inclined plane outside the coxite there is a small protrusion that should be the stylus because the valvifer connects with the coxite and paraproct at the base. This structure is similar to the membrane of the ovipositor in the Camptosomata (especially in some species in the genera *Cryptocephalus* and *Clytra*). Both the Synetinae and Camptosomata have a well-developed proctiger and a broad, sclerotized paraproct. Based on similar oviposition structure and behaviors, we speculate that the Synetinae has the closest relationship to the Camptosomata in the Chrysomelidae. However, we cannot exclude the possibility of parallel or convergent evolution of oviposition behavior in both taxa. Schmitt (1996) suggested that the Camptosomata, Synetinae, and some species in the Eumolpinae belonged to a monophyletic group because their Kotpresses were homologous structures. Our observation supports Schmitt (1996), but we found that the ovipositor of the Eumolpinae is ancestral and differs from the ovipositor in the Camptosomata. We have not observed the egg-laying habits of the genus *Chlamisus*.

**VII.** Based on comprehensive observations of the female genitalia of the Chrysomeloidea, it was found that the apices of the ovipositor are usually palp-like and backward except in the subfamily Cassidinae and in the genus *Callispa* of Hispini, which has a wide, plate-like ovipositor. Muir and Sharp (1904) found that the plate-like ovipositor of *Aspidomorpha puncticosta* Boheman was the key structure required to form the diaphragm between the eggs in the egg sac. Chen et al. (1986) suggested that the trait of laying eggs in an egg sheath in the genus *Callispa* and subfamily Cassidinae was further evidence of their close relationship. Our studies show that the plate-like ovipositor is found only in species that make a sheath to protect an egg. In addition, we found that the ovipositor of the genus *Leptispa* belongs to the same tribe as the genus *Callispa* and it is palp-like, not plate-like. We also found that the adult and larvae in the genus *Leptispa* live in a leaf roll (Chen 1986). The adult lays its egg in a leaf roll but does not make a sheath. Therefore we suggest that the morphology of the ovipositor in these genera show a close relationship even though they differ in life-history. Based on this evidence, we conclude that the genus *Callispa* branched early and the genus *Leptispa* appeared later.

## Conclusions

Female genitalia are diverse, complex, and very important for classification of the Chrysomeloidea. With a comparative analysis of the female genitalia we can reach a well-resolved classification system of this family. Egg-laying behavior and oviposition media cannot to be ignored when we explore the evolution of this huge taxon, but we still know very little about the reproductive biology of individual species. This research is particularly limited in Chinese lineages and future research is desperately needed to compare all the members of this lineage to resolve the systematics of the Chrysomeloidea.
